# The General Hospital, Birmingham

**Published:** 1893-11-04

**Authors:** 


### THE GENERAL HOSPITAL, BIRMINGHAM.

Mr. Howard J. Collins, House Governor, writes under date October 24th: "In your article 'Hospital Administration,' on page 45 of last week's issue of The Hospital, you place this hospital at the head of the list of provincial hospitals, with a percentage of 10·460 cost of administration to maintenance, and 9·422 administration to total ordinary expenditure. This, it is clear, arises from the fact that in the last published accounts the items for administration and maintenance are not shown separately, and, as stated in my letter to the editor of the *Hospital Annual* on the 6th March last, and again in the return sent to him on the 14th September, I pointed out that the salaries of officers in the report includes the resident medical and surgical officers, chaplain, pathologist, matron, and housekeeper; and under stationery, advertising, printing, postages, &c., the whole cost of these articles.

"Having now adopted the Uniform System of accounts in this hospital, our next published report and balance-sheet will show the details of maintenance and administration, which will enable you and others to compare it with other institutions, but in the meantime I trust you will do us the justice of correcting the figures in your article above referred to. They should be: total expenditure £15,596 11s. 5d.; management, £1,196 7s. 5d.; maintenance, £14,400 4s.; giving percentages of 8·305 to maintenance, and 7·668 to total expenditure."

*** We rejoice to know that the Birmingham General Hospital has wisely determined to keep its accounts on the Uniform System. The necessity for this is clearly shown by the above letter. Mr. Collins is in error in supposing that the difference between the percentages he quotes and those given in the table on page 45 of The Hospital for October 21st, 1893, are due to the causes mentioned by him. In appearance they may be so, but, in fact, the differences arise from the difficulty of apportioning the miscellaneous items set forth in the accounts, and the inclusion of the cost of expenditure upon convalescent institutions. The adoption of the Uniform System of accounts—a course which all the best hospitals are now taking—will at last enable true comparisons to be drawn upon identical figures.—Ed. *T. H.*

